# Universal Precautions Plus: Physician-Directed Strategies for Improving Patient Health Literacy in the Emergency Department

**DOI:** 10.5811/westjem.2022.10.57697

**Published:** 2022-12-28

**Authors:** Jamaji C. Nwanaji-Enwerem, Mikhaila Smith-Wilkerson, Brittney Gordon, Helene Okpere, Terrell Jones, Rahel Gizaw, Irfan Husain

**Affiliations:** * Emory University School of Medicine, Department of Emergency Medicine, Atlanta, Georgia; † Emory Rollins School of Public Health, Gangarosa Department of Environmental Health, Atlanta, Georgia

## Abstract

Working on the frontlines with safety-net populations, emergency physicians are uniquely positioned to take on a greater role in addressing the current health literacy crisis and specific barriers that may exist. Here, we review the concept of universal health literacy precautions and explore the application of these universal precautions in conjunction with other patient-centered strategies. More specifically, to improve patient understanding and outcomes, emergency physicians can pair universal health literacy precautions with strategies including multiple learning techniques, dual-code theory, empowerment counseling, family buy-in, and hands-on practice. We provide two examples of emergency department encounters where this combined approach was used differently yet successfully and efficiently. Ultimately, we aim to highlight the value of emergency physicians being equipped with basic skills in health literacy educational strategies.

## INTRODUCTION

Patient health literacy should be an important consideration for all physicians because of its relationship to important health outcomes. Low health literacy has been associated with lower utilization of preventive services, lower rates of medication adherence, greater numbers of hospitalizations, poorer overall health, and higher rates of mortality.^1^,^2^ Low patient health literacy is particularly important for emergency physicians because it is a significant contributor to higher uses of emergency care,^3^ including emergency department (ED) visits for complaints that could otherwise be addressed in an ambulatory context^4^ and ED visits that result in hospitalizations.^5^ In short, health literacy is a problem for both non-acute and acute patients. Furthermore, because many patients initially, or most frequently, interact with the healthcare system through the ED, emergency physicians are uniquely positioned to address health literacy concerns.

### Defining Health Literacy

The United States (US) Department of Health and Human Services and the Institute of Medicine define health literacy as “the degree to which individuals have the capacity to obtain, process, and understand basic health information and services needed to make appropriate health decisions.”^6^ This definition focuses on clinical risk and is framed as an individual deficit. To emphasize the role of personal as well as social and environmental determinants of health, it has been proposed that health literacy be framed as an asset to be built.^7^ The asset framework further emphasizes the co-creation of knowledge between physicians and patients while prioritizing patient preferences and values.^8^ Combining both the risk and asset frameworks results in a comprehensive definition of health literacy including functional, interactive, and critical thinking skills.^7^

Functional skills include the basic ability to write and read information such as medication labels. Interactive skills include more advanced literacy and cognitive skills that enable patients to understand and apply medical information in ever-changing circumstances. At this level of health literacy, a patient may obtain information from a medication advertisement and can discuss their interest or concerns about the advertisement while creating a care plan with their physician. Critical literacy skills are even more advanced and include the ability to critically use information to implement lifestyle and living condition changes when possible. Patients with critical literacy demonstrate the greatest effectiveness in making informed decisions and in health self-management. This includes comfort in knowing when to ask for assistance in decision-making.^7^

### The Emergency Department and Universal Precautions Plus

As with other health-related experiences, EDs remain an important population safety net. Thus, emergency physicians must be equipped with skills to identify and approach health literacy concerns effectively and in a manner that is efficient given their working environment. In 2003, the US Department of Education conducted a national assessment of health literacy skills – measuring the ability to read health-related information and manage numerical information related to health – and found that 88% of US adults do not have the health literacy skills needed for handling the demands of the current healthcare system.^6^ Given this finding and research reporting that clinicians have difficulty identifying patients with low health literacy,^9^ professional organizations recommend using universal health literacy precautions to improve accessibility of health information to all patients regardless of education or literacy levels.^9^,^10^

Universal health literacy precautions aim to simplify written and verbal communication, confirm comprehension using “teach-back,” improve navigation of the healthcare system, and empower patients’ efforts to improve their health.^10^ Although universal health literacy precautions help in increasing the accessibility of the information that patients receive,^9^ a prior ED randomized trial reported that patients demonstrated even greater learning when they also received materials matched to their preferred learning styles.^2^ However, there has been controversy surrounding learning styles and data demonstrating that learning styles have no bearing on individuals’ ability to learn and retain material.^11^ Still, this may not mean that recognizing learning styles is completely futile. In fact, it has been proposed that learning styles may be of limited utility given their prescriptive nature; however, reframing them as learning strategies or techniques allows for more flexibility and allows learners to use a variety of styles depending on the task at hand.^12^ Given this data, to aid efforts directed at educating emergency physicians in building a more robust health literacy skillset we provide examples of ED patient encounters where additional strategies were paired with universal health literacy precautions and multiple learning techniques to successfully improve patient health literacy.

### Dual Coding and Hands-On Practice to Address “Noncompliance” in Respiratory Failure

A bedbound, 48-year-old man with a past medical history of pulmonary hypertension, obstructive sleep apnea, and heart failure presented to the ED with confusion. He received an extensive infectious and metabolic work-up including an arterial blood gas and computed tomography imaging of the brain. Given initial labs showing that he was acidotic and hypercapnic, he was placed on bilevel positive airway pressure, which normalized his pH and mental status. Nevertheless, his arterial carbon dioxide levels remained slightly elevated, resulting in our belief that his presentation likely reflected an acute-on-chronic process. As the patient’s mental status improved, he revealed to the treatment team that he had not been using his continuous positive airway pressure (CPAP) machine at home. He continued to explain to the team that he had significant difficulty and discomfort with putting on the CPAP mask due to his body habitus, and his caretakers were not able to assist him at night. Throughout the conversation, the patient admitted that when he was initially prescribed CPAP, he did not understand his need for it or how it would help him.

Understanding that properly delivered patient education has improved outcomes in patient compliance, satisfaction, and healthcare utilization,^13^ we appreciated that this man’s future health depended on the education provided at this visit. We also recognized that each patient has a unique cognitive load or working memory that represents their capacity to receive and process finite amounts of information.^14^,^15^ In agreement with the use of flexible learning techniques, the team aimed to maximize the patient’s uptake of information through Allan Paivio’s dual-code theory.^16^ Dual-code theory acknowledges both verbal and non-verbal cognitive processing and then leverages words and imagery to improve patient memory and enhance learning.^17^ Different from a prescriptive learning style, this approach places a significant and equal emphasis on verbal and non-verbal modalities of education.

We planned to use two separate working memory systems – visual (non-verbal) and auditory – to present complex medical diagnosis and management information in a manner that would not cause cognitive overload. Along with verbal teaching, we used simple visual aids to explain the etiology of the patient’s symptoms and how CPAP can prevent future episodes of this initial presentation to the ED. After the education, the patient was able to demonstrate understanding of the information through “teach-back.” Furthermore, given that the patient previously expressed difficulty with placing his CPAP mask, we asked our respiratory therapist to spend some time with him to fit his mask and practice mask placement and removal. Review of tasks is likely helpful to all patients, taps into the domain of kinesthetic learning, and further reinforces previous non-verbal efforts employed in dual coding.^2^

### Empowerment Counseling and Family Buy-In to Address Disinformation in Leg Pain

A 68-year-old woman with a past medical history of type II diabetes and degenerative disc disease treated with lumbar spinal fusion surgery six years prior presented to the ED for bilateral shooting leg pain that had been ongoing for several months. The patient reported seeing an outpatient pain management doctor during the prior year who discontinued her gabapentin because it “was one of the worst medications on the market and would cause her legs to become necrotic and fall off.” Instead, she was told that she needed a $3,000 procedure to manage her pain. In the ED, her vital signs were within normal limits. She stated that her pain was tolerable, but she was amenable to acetaminophen while we completed her evaluation. Her physical exam was unremarkable including no spinal tenderness. Gross motor and sensation were intact, and she had a negative bilateral straight leg test. She was able to ambulate without deficits. Her lab work demonstrated no electrolyte disturbances.

Upon completion of her evaluation, we believed that her symptoms were most likely due to neuropathic pain that would benefit from gabapentin. However, we knew that she would be apprehensive about restarting gabapentin due to the fear of losing her legs. With respect to her specific health literacy barriers, we needed to address the gabapentin disinformation. She expressed a preference for visual materials. Thus, embracing the techniques of dual coding and using multiple, flexible learning techniques, we tapped into the visual/non-verbal domain by printing easy-to-understand tables and figures describing gabapentin side effects – none of which were limb necrosis.^18^ We also focused on the verbal/auditory domain and, at the bedside, reviewed these visual materials using positive and empowerment counseling techniques shown to be effective from the musculoskeletal pain literature.^8^

These techniques involve avoiding low recovery expectations, promoting the attempted resumption of daily activities even when still experiencing pain, and emphasizing the improvement of activity levels as an important endpoint – not simply pain relief. When available, other useful auditory/verbal tools include video discharge instructions and follow-up caseworker phone calls.^18^ During our discussion, the patient’s adult son arrived in the room and we were also able to share the information with him. This was critical as family involvement has been identified as a cornerstone for successful shared decision-making with elderly patients.^19^ Ultimately, the patient agreed to restart her gabapentin and was given information to schedule a primary care visit with a physician at our institution. Review of her chart demonstrated that she made her follow-up visits and reported improvement in her pain symptoms.

### Building Health Literacy Skills

If not doing so already, all physicians – especially those in the ED – should be using the basics of universal health literacy precautions. Nevertheless, we recognize that like many skills in medicine, the strategies for improving patient health literacy do not often come automatically to physicians and require training, particularly in ED encounters where physician-patient interaction time is limited.^2^,^20^ Resources like the Agency for Healthcare Research and Quality’s Health Literacy Universal Precautions Toolkit can be implemented in curricula at the medical student and resident level to aid in bolstering educational efforts in this area.^10^ There is also an opportunity to practice health literacy skills in simulation-based education delivered as part of didactics. Importantly, health literacy should be viewed as a dynamic concept.

Different learning tools will have different efficacies depending on the patient. Even for one individual, the dynamism of his/her/their own health status can result in new deficits in their health literacy.^21^ Simultaneously, clinical understanding of disease process and management schemes is also ever evolving. Thus, physicians will have to work at maintaining their health literacy skills throughout their careers. Pursuing formal health literacy activities for continuing medical education credit may prove helpful in this endeavor.

## CONCLUSION

**Figure 1:**
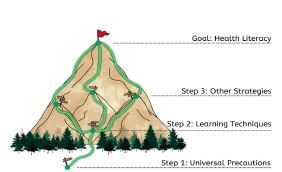
Approach for improving patient health literacy. The paths for improving patient health literacy are vast. We recommend beginning with universal health literacy precautions. We then recommend using a multi-learning technique strategy such as dual coding followed by the application of other strategies (eg, positive empowerment counseling, family-involved shared decision-making, hands-on medical equipment skills practice, etc).

While on the job, emergency physicians must actively remain vigilant to identify instances where addressing health literacy may require more than the universal precautions. Important warning signs may include frequently missed appointments, incomplete registration forms, medication noncompliance, or even a patient who does not ask many questions during the medical encounter.^22^ Given the gravity of health literacy to individual and population health, we provide these strategies (eg, the application of multiple learning techniques/dual coding, positive empowerment counseling, family-involved shared decision-making, hands-on medical equipment skills practice, etc.) in addition to universal health literacy precautions to bolster emergency physicians’ chances of improving patient understanding and thereby health outcomes (Figure 1). 21
